# Hartung-Knapp-Sidik-Jonkman approach and its modification for random-effects meta-analysis with few studies

**DOI:** 10.1186/s12874-015-0091-1

**Published:** 2015-11-14

**Authors:** Christian Röver, Guido Knapp, Tim Friede

**Affiliations:** Department of Medical Statistics, University Medical Center Göttingen, Humboldtallee 32, Göttingen, 37073 Germany; Department of Statistics, TU Dortmund University, Dortmund, 44221 Germany

**Keywords:** Random-effects meta-analysis, Knapp-Hartung adjustment, Small populations, Rare diseases

## Abstract

**Background:**

Random-effects meta-analysis is commonly performed by first deriving an estimate of the between-study variation, the *heterogeneity*, and subsequently using this as the basis for combining results, i.e., for estimating the *effect*, the figure of primary interest. The heterogeneity variance estimate however is commonly associated with substantial uncertainty, especially in contexts where there are only few studies available, such as in small populations and rare diseases.

**Methods:**

Confidence intervals and tests for the effect may be constructed via a simple normal approximation, or via a Student-*t* distribution, using the Hartung-Knapp-Sidik-Jonkman (HKSJ) approach, which additionally uses a refined estimator of variance of the effect estimator. The modified Knapp-Hartung method (mKH) applies an *ad hoc* correction and has been proposed to prevent counterintuitive effects and to yield more conservative inference. We performed a simulation study to investigate the behaviour of the standard HKSJ and modified mKH procedures in a range of circumstances, with a focus on the common case of meta-analysis based on only a few studies.

**Results:**

The standard HKSJ procedure works well when the treatment effect estimates to be combined are of comparable precision, but nominal error levels are exceeded when standard errors vary considerably between studies (e.g. due to variations in study size). Application of the modification on the other hand yields more conservative results with error rates closer to the nominal level. Differences are most pronounced in the common case of few studies of varying size or precision.

**Conclusions:**

Use of the modified mKH procedure is recommended, especially when only a few studies contribute to the meta-analysis and the involved studies’ precisions (standard errors) vary.

## Background

Random-effects meta-analysis is most commonly performed based on an underlying hierarchical model including two unknowns as parameters: the *effectμ*, which is the figure of primary interest, and the *between-study variance (heterogeneity)τ*^2^, which is a nuisance parameter. Inference then is usually done sequentially, by first deriving an estimate of the heterogeneity variance, $\hat {\tau }^{2}$, and then determining the effect estimate $\hat {\mu }$ by conditioning on the estimate $\hat {\tau }^{2}$ [1, 2]. A large number of different estimators for the heterogeneity variance is available (see e.g. [3–6]), and effect estimation may be done based on a simple normal approximation, or by utilizing a Student-*t* distribution [7] with an additionally refined estimator of the variance of $\hat {\mu }$ [8–12]. While the normal model may be motivated by asymptotic arguments, in actual applications the number of estimates to be combined is commonly small [13, 14] and hence the estimation uncertainty in the between-study variance *τ*^2^ is substantial, so that an adjustment is appropriate and in fact improves operating characteristics [7–12, 15].

The problem of deriving estimates from only a small number of data sources is a common problem especially in fields of application where empirical information is sparse due to the rarity of the condition in question. The rarity of a disease is often accompanied with a low (commercial) interest or incentive, which is why such diseases are also known as *orphan diseases*. According to the European Commission, a disease is designated orphan status when the prevalence is ≤5 in 10 000 [16]. While by definition any individual rare disease has a low prevalence, there is a large number of these, eventually affecting a substantial fraction of an estimated 6–8 % of the total population [17], and with that posing a challenge to health care systems worldwide.

The European Medicines Agency acknowledges the particular obstacles in rare diseases research but points out that there is no fundamental difference between rare and more common diseases and hence no “paradigm change” when it comes to regulatory issues. Because of the common small-sample settings, the importance of sophisticated methods is emphasized, and meta-analyses of good quality randomised controlled clinical trials are still considered the highest level of evidence [18]. The problems encountered in rare diseases research often call for special statistical methods, especially with respect to study designs [17, 19, 20]. Meta-analyses are particularly important in this field due to the lack of large trials, while these will commonly still be faced with the problem of small numbers of available studies. Between-study heterogeneity then is anticipated, since the gathered pieces of evidence are likely to differ with respect to study designs, types of control groups or treatment allocation [17, 19, 20], Unkel S, Röver C, Stallard N, Benda N, Posch M, Zohar S, et al., Systematic reviews in paediatric multiple sclerosis and Creutzfeldt-Jakob disease exemplify shortcomings in methods used to evaluate therapies in rare conditions, Submitted. Small studies have in fact epirically been found to exhibit more heterogeneity than large trials [21]. Consequently, the use of methods suitable for few studies and marginally significant findings is of crucial importance here.

With an estimated incidence of 2–20 cases per 100 000 population, juvenile idiopathic arthritis (JIA) is an example of a rare disease [22]. In the following, we will use a meta-analysis in JIA [23] as a case study to illustrate the different methods discussed below.

In the following sections, we will first describe the methods used, then show the results of a simulation study, and demonstrate the different types of analyses in an example data set, before closing with conclusions and recommendations.

## Methods

### Random-effects meta-analysis

Meta-analysis is very commonly performed via a *random-effects* approach, utilizing the *normal-normal hierarchical model*. Here the data are given in terms of a number *k* of estimates *y*_*i*_∈*ℝ* that are associated with some uncertainty given through standard errors *s*_*i*_>0 that are taken to be known without uncertainty. The estimates are assumed to measure trial-specific parameters *θ*_*i*_∈*ℝ*:

(1) $$ y_{i} \sim \mathrm{N}\left(\theta_{i}, {s_{i}^{2}}\right) \qquad \text{for}\, i = 1,\ldots,k.  $$

The parameters *θ*_*i*_ vary from trial to trial around a global mean *μ*∈*ℝ* due to some *heterogeneity variance* between trials that constitutes an additive variance component to the model,

(2) $$ \theta_{i} \sim \mathrm{N}\left(\mu, \tau^{2}\right) \qquad \text{for}\, i = 1,\ldots,k,  $$

where *τ*^2^≥0. The model may then be simplified by integrating out the parameters *θ*_*i*_, leading to the marginal expression

(3) $$ y_{i} \sim \mathrm{N}\left(\mu,\, {s_{i}^{2}} + \tau^{2}\right) \qquad \text{for}\, i = 1,\ldots,k.  $$

Among the two unknowns in the model, the overall mean *μ*, the *effect*, usually is the figure of primary interest, while the heterogeneity variance *τ*^2^ constitutes a nuisance parameter. When *τ*^2^=0, the model simplifies to the so-called *fixed-effect* model [1, 2].

The “relative amount of heterogeneity” in a meta-analysis may be expressed in terms of the measure *I*^2^, which is defined as

(4) $$ I^{2} = \frac{\tau^{2}}{\tau^{2}+\tilde{s}^{2}}  $$

where $\tilde {s}$ is some kind of “average” standard error among the study-specific *s*_*i*_ [24]. In the following simulation studies, we will determine $\tilde {s}^{2}$ as the arithmetic mean of squared standard errors.

### Parameter estimation

If the value of the heterogeneity variance parameter *τ*^2^ were known, the (conditional) maximum-likelihood effect estimate would result as the weighted average

(5) $$ \hat{\mu} = \frac{\sum_{i} w_{i} \,y_{i}}{\sum_{i} w_{i}}  $$

with “inverse variance weights” defined as

(6) $$ w_{i} = \frac{1}{{s_{i}^{2}} + \tau^{2}} \qquad \text{for} \,i = 1,\ldots,k.  $$

A common approach to inference within the random-effects model is to first estimate the heterogeneity variance *τ*^2^, and subsequently estimate the effect *μconditional on the heterogeneity estimate*. Note that the *w*_*i*_ are effectively treated as “known” while in fact both the ${s_{i}^{2}}$ as well as *τ*^2^ are only measured with some uncertainty (that depends on the size/precision of the *i*th individual study and the number of studies *k*). There is a wide range of different heterogeneity estimators available (see e.g. [3–6] for more details). In the following we will concentrate on some of the most common ones, the DerSimonian-Laird (DL) estimator, a moment estimator [25] with acknowledged shortcomings [26], the restricted maximum likelihood (REML) estimator [3, 27], and the Paule-Mandel (PM) estimator, an essentially heuristic approach [28, 29]. Software to compute the different estimates is provided e.g. in the metafor and metaR packages [30, 31].

### Confidence intervals and tests

#### Normal approximation

Confidence intervals and, equivalently, tests for the effect *μ* are commonly constructed using a normal approximation for the estimate $\hat {\mu }$. The standard error of $\hat {\mu }$, conditional on a fixed heterogeneity variance value *τ*^2^, is given by

(7) $$ \hat{\sigma}_{\mu} = \sqrt{\frac{1}{\sum_{i} w_{i}}}.  $$

A confidence interval for the effect *μ* then results via a normal approximation as

(8) $$ \hat{\mu} \; \pm \; \hat{\sigma}_{\mu} \; z_{(1-\alpha/2)}  $$

where *z*_(1−*α*/2)_ is the (1 − *α*/2)-quantile of the standard normal distribution, and (1 − *α*) is the nominal coverage probability [1, 2]. The normal approximation based on a heterogeneity variance estimate $\hat {\tau }^{2}$ usually works well for many studies (large *k*) and small standard errors (small *s*_*i*_), or negligible heterogeneity (small variance *τ*^2^), but tends to be anticonservative otherwise [8, 9], Friede T, Röver C, Wandel S, Neuenschwander B, Meta-analysis of few small studies in orphan diseases, Submitted.

#### The Hartung-Knapp-Sidik-Jonkman (HKSJ) method

Hartung and Knapp [8, 9] and Sidik and Jonkman [10] independently introduced an adjusted confidence interval. In order to determine the adjusted interval, first the quadratic form

(9) $$  q = \frac{1}{k-1} \sum_{i} w_{i}(y_{i}-\hat{\mu})^{2}  $$

is computed [8–10]. The adjusted confidence interval then results as

(10) $$ \hat{\mu} \; \pm \; \sqrt{q} \; \hat{\sigma}_{\mu} \; t_{(k-1); (1-\alpha/2)}  $$

where *t*_(*k*−1);(1−*α*/2)_ is the (1 − *α*/2)-quantile of the Student-*t* distribution with (*k* − 1) degrees of freedom. Note that $q \hat {\sigma }^{2}\mu $ is derived from a non-negative and unbiased estimator of $\frac {1}{\sum _{i} w_{i}}$ [32]. Confidence intervals based on the normal approximation may easily be converted to HKSJ-adjusted ones [12]. The method has also been generalized to the cases of multivariate meta-analysis and meta-regression [33].

#### The modified Knapp-Hartung (mKH) method

The HKSJ confidence interval (10) tends to be wider than the one based on the normal approximation (8), since the Student-*t* quantile is larger than the corresponding normal quantile, while *q* will tend to be somewhere around unity. However, *q* may in fact also turn out arbitrarily small, and if $\sqrt {q}<\frac {z_{(1-\alpha /2)}}{t_{(k-1);(1-\alpha /2)}}$, then the modified interval will be shorter than the normal one, which may be considered counter-intuitive. A simple *ad hoc* modification to the procedure results from defining

(11) $$  q^{\star} = \max\{1, q \}  $$

[11] and using *q*^⋆^ instead of *q* to construct confidence intervals and tests. This will ensure a more conservative procedure. The modification was originally proposed in the meta-regression context, but the simple meta-analysis here constitutes the special case of an “intercept-only” regression.

Note that the PM heterogeneity variance estimator is effectively defined by choosing $\hat {\tau }^{2}$ such that *q* (Eq. (9)) is = 1 (or less, if no solution exists) [28, 29], so that for the PM estimator the corresponding *q*^⋆^ value always equals *q*^⋆^ = 1.

### Simulations

Since a (1 − *α*) confidence interval is supposed to cover the true parameter value with probability (1 − *α*), the calibration of such intervals may be checked by repeatedly generating random data based on known parameter values and then determining the empirical frequency with which true values are actually covered [34]. We performed such a *Monte Carlo* simulation comparing the HKSJ and mKH approaches using the setup that was introduced by IntHout et al. [12]. Data were simulated on a continuous scale, according to the random-effects model described above, with study-specific standard errors *s*_*i*_ set to reflect certain scenarios with respect to the relative size of studies and their variation due to estimation uncertainty. Many meta-analyses are based on (discrete) count data, where the random-effects model assumptions only hold to an approximation that works well unless event probabilities are very low. Alternative methods have been proposed to deal with low event probabilities [35, 36], but low-event-rate effects were not considered in the present investigation. These simulations considered four different scenarios, namely meta-analyses (A) with trials of equal size, (B) with equally sized trials but including one small trial, (C) with 50 % large and small trials, and (D) equally sized trials and one large trial. The sizes of “small” and “large” trials (and hence, squared standard errors ${s_{i}^{2}}$) here differ by a factor of ten, so that the associated standard errors differ by roughly a factor of 3; for more details see ([12], Appendix 2). Numbers of studies *k* considered here are in the range of 2–11, and the true levels of heterogeneity were *I*^2^∈{0.00,0.25,0.50,0.75,0.90}. At each combination of parameters 10 000 meta-analyses were simulated. All simulations were performed using R [37].

## Results

### Simulations

**Fig. 1:**
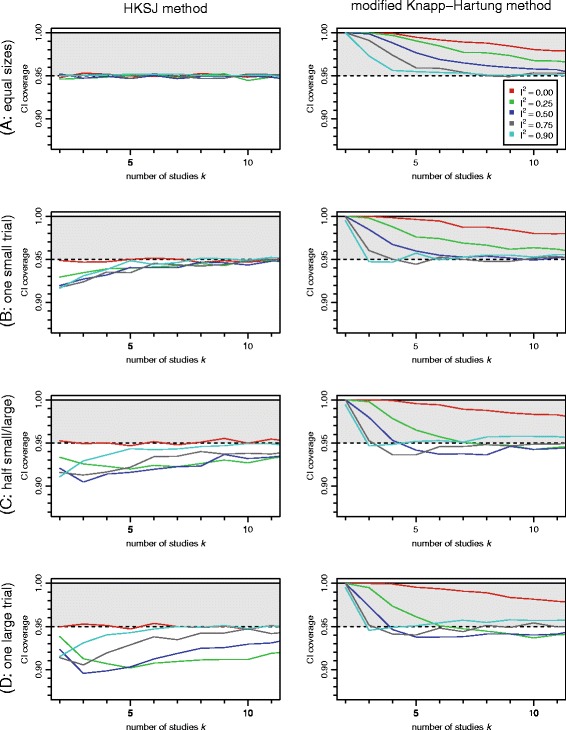
Coverage probabilities of HKSJ and mKH 95 % confidence intervals. Probabilities are shown in dependence of the number *k* of studies and the amount of heterogeneity *I* ^2^. The four different scenarios A–D correspond to different amounts of imbalance between the study-specific standard errors *s*_*i*_. The DerSimonian-Laird (DL) method was used for estimation of the heterogeneity variance *τ* ^2^

Figure 1 shows the estimated coverage probabilities of the different confidence intervals based on the DL heterogeneity variance estimate. The corresponding figure for REML and PM look essentially the same, which is in line with the findings in [38], Friede T, Röver C, Wandel S, Neuenschwander B, Meta-analysis of few small studies in orphan diseases, Submitted. The HKSJ method works very well when the analyzed studies are of equal size (i.e., have equal standard errors), as can also be shown analytically [39], but coverage decreases in more imbalanced settings, especially for small numbers of studies. For the case of no heterogeneity (*I*^2^=0) the HKSJ method also works fine, but if *τ*^2^ was known a priori, in this case the fixed-effect model should work as well. The mKH procedure on the other hand is rather conservative for small *k*, but does not tend to inflate the type-I error substantially regardless of the underlying study sizes or true heterogeneity. For both methods, the dependence on the amount of heterogeneity is mostly a matter of whether *τ*^2^ is =0 or >0.

**Fig. 2:**
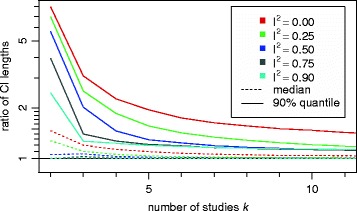
Ratios of lengths of HKSJ and mKH confidence intervals. (Same as $\frac {\sqrt {q^{\star }}}{\sqrt {q}}$). Lengths are shown in dependence of the number *k* of studies and the amount of heterogeneity *I* ^2^. Numbers are averaged over all 4 scenarios. The DL method was used for *τ* ^2^ estimation

Application of the modification obviously tends to widen the resulting confidence intervals. The ratio of interval lengths (which is equal to $\frac {\sqrt {q^{\star }}}{\sqrt {q}}$) is shown in Fig. 2. Most notably, the effect is largest for small heterogeneity and for few studies.

The modification eventually only makes a difference in those cases where *q* turns out smaller than one. The fraction of intervals affected by the modification ranges between 31 % and 82 % for the DL heterogeneity variance estimator and for the scenarios investigated here, with an overall average of 61 %. For the other two estimators the fractions are 29–82 % with a mean of 62 % (REML), and 31–91 % with a mean of 74 % (PM). Again, the differences between the different estimators are rather small. With respect to the underlying simulation scenario, the probability decreases with increasing heterogeneity, since larger heterogeneity also leads to larger values of *q*.

### Application to JIA example data

**Fig. 3:**
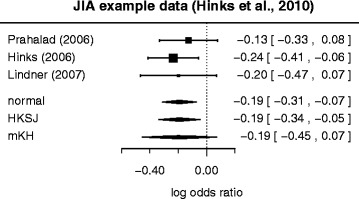
Hinks et al. (2010) example data. A forest plot illustrating the three estimated log odds ratios and 95 % confidence intervals for the example data due to Hinks et al. [23]. The estimated amount of heterogeneity variance (according to DL, REML and PM estimators) is zero here. At the bottom the three alternative combined estimates based on normal approximation, HKSJ and mKH approach are shown

Hinks et al. [23] studied the occurrence of a particular genetic variant, CCR5, in juvenile idiopathic arthritis (JIA) patients in comparison with the general population. Their investigation included a meta-analysis of a small number (*k*=3) of available controlled studies looking into the association of JIA with this particular biomarker. The analysis was based on logarithmic odds ratios; the three estimates along with their standard errors are shown in a forest plot in Fig. 3. Here the largest standard error is 50 % larger than the smallest one. For these data, all three (DL, REML and PM) heterogeneity variance estimates turn out as $\hat {\tau }^{2}\,=\,0$. A zero heterogeneity variance estimate is not uncommon, even when the actual heterogeneity is in fact substantial [14]. The associated *q* value is also small with *q*=0.31 ($\sqrt {q}=0.55$). The resulting three confidence intervals based on normal approximation, HKSJ adjustment and mKH method all differ in their lengths. The mKH interval is longest, and includes the zero log odds ratio (indicating no association between genetic marker and disease), while the other two intervals do not include zero. So the choice of procedure directly affects conclusions in this example.

In this example, the three standard errors are rather similar (the largest is 50 % larger than the smallest), but while the heterogeneity variance estimates $\hat {\tau }^{2}$ are all at zero, the 95 % Q-profile confidence interval [40] ranges up to *τ*^2^=0.33^2^, corresponding to *I*^2^=0.90. Within the context of our simulations (Fig. 1), we cannot tell from the data which of the heterogeneity (*I*^2^) scenarios we are in fact in, and as the standard errors are not exactly the same, it remains a matter of balancing the potential consequences whether one would rather risk losing on the side of (type I) error probability or power.

## Conclusions

The HKSJ procedure ensures the coverage probability only when the included studies’ standard errors *s*_*i*_ are similar; for unbalanced settings, the actual error probability tends to exceed the targeted one. With the standard definition of the correction factor *q* the results may sometimes be counterintuitive, since the corresponding CIs may turn out shorter than using the simple normal approximation; in fact they may get arbitrarily short. In case of no heterogeneity (*τ*=0) the HKSJ method also works well, however practically this is of limited relevance, as one can rarely tell (or convincingly argue) whether this condition holds.

The *ad hoc* modification of the mKH method aims at fixing these shortcomings and results in type-I error probabilities that are not grossly in excess of the pre-specified ones. Especially when the standard errors *s*_*i*_ are of dissimilar magnitude, the mKH method can therefore be recommended. For few studies (small *k*), the modified procedure however tends to be very conservative, with very small error probabilities especially in the extreme case of meta-analysis of only *k* = 2 studies. In this extreme case the choice of methods may therefore be considered a matter of a power vs. type-I error probability tradeoff.

While meta-analyses of few studies are a particular problem in indications where there is only little evidence available (such as rare diseases), such circumstances are not as uncommon as one might expect. Turner et al. [13] and Kontopantelis et al. [14] investigated the analyses archived in the Cochrane Database and actually found a *majority* of them to be based on as few as *k* = 2 or *k* = 3 studies; so these constitute highly relevant cases for which the proper control of error rates is crucial.

The properties of either unmodified or modified method for the extreme case of *k* = 2 may be considered unsatisfactory, as it seems one has the choice of either falling short of or exceeding the targeted error probability; the problem has in fact been regarded as effectively unsolved [41]. The poor behaviour may be explained by the fact that performing a random-effects meta-analysis effectively means the estimation of first- and second-order statistics, and it is not overly surprising to find that this is a hard task when the data consist of as few as two samples that are only measured with uncertainty. Bearing this in mind, the use of Bayesian methods [42] and the consideration of external evidence on the likely magnitude of the heterogeneity [43] may be the way forward.

## Abbreviations

DL: DerSimonian-Laird
HKSJ: Hartung-Knapp-Sidik-Jonkman
JIA: juvenile idiopathic arthritis
mKH: modified Knapp-Hartung
PM: Paule-Mandel
REML: restricted maximum likelihood
